# Evaluation of the Wound Healing Potential of *Resina Draconis (Dracaena cochinchinensis)* in Animal Models

**DOI:** 10.1155/2013/709865

**Published:** 2013-05-16

**Authors:** Huihui Liu, Shaohui Lin, Dan Xiao, Xiao Zheng, Yan Gu, Shanyu Guo

**Affiliations:** ^1^Department of General Surgery, Ninth People's Hospital, Shanghai Jiao Tong University School of Medicine, Shanghai 200011, China; ^2^Department of Geriatrics, Ninth People's Hospital, Shanghai Jiao Tong University School of Medicine, Shanghai 200011, China

## Abstract

*Resina Draconis* (RD) is a type of dragon's blood resin obtained from *Dracaena cochinchinensis* (Lour.) S.C. Chen (Yunnan, China). It has been used as a medicine since ancient times by many cultures. The ethanolic extract of *Resina Draconis* (RDEE) was evaluated for its wound-healing activity using excision and incision wound models in rats. Group I, the control group, was treated with ointment base. Group II, which served as a reference standard, was treated with moist exposed burn ointment (MEBO). Group III was treated with RDEE. The parameters observed were percentage of wound contraction, epithelialization period, tensile strength, histopathological studies, microvessel density (MVD), and the expression of vascular endothelial growth factor (VEGF) and transforming growth factor-**β**1 (TGF-**β**1). The group treated with RDEE showed significantly better wound contraction and better skin-breaking strength as compared with the control group. The results of histopathological examination, MVD, and the expression levels of growth factors supported the outcome of the wound models as well. The present study provided a scientific rationale for the traditional use of RD in the management of wounds.

## 1. Introduction

Skin healing is a complex process that involves inflammation, reepithelization, angiogenesis, granulation tissue formation, and deposition of interstitial matrix, beside other events carried out by different types of cells, such as keratinocytes, fibroblasts, inflammatory cells, and endothelial cells. There are three stages of the process of wound healing: inflammation, proliferation, and remodeling. The proliferative phase is characterized by angiogenesis, collagen deposition, granulation tissue formation, epithelization, and wound contraction. Angiogenesis involves new blood vessel growth from endothelial cells. In fibroplasia and granulation tissue formation, fibroblasts excrete collagen and fibronectin to form a new extracellular matrix. Subsequently epithelial cells crawl across the wound bed to cover it and the wound is contracted by myofibroblasts, which grip the wound edges and undergo contraction using a mechanism similar to that in smooth muscle cells. The final stage of wound healing is remodeling or maturation of the granulation tissue into mature connective tissue and/or scar. Alterations in any of these steps can lead to healing delay or even the inability to heal completely [1]. Current methods used to treat wounds include debridement, irrigation, antibiotics, tissue grafts, and proteolytic enzymes, which possess major drawbacks and unwanted side effects. The use of traditional medicinal remedies and plants in the treatment of burns and wounds is an important aspect of health management and at the same time is an effective way to provide cheaper healthcare options [2].

Since ancient times, people have used plants and preparations thereof to accelerate the wound-healing process [3]. Recently, the interest of using alternative therapies and natural remedies in wound management has rapidly increased. There are hundreds of medicinal plants that have long histories of curative properties against various diseases and ailments. However, their use is merely based on tradition, without any scientific evidence of their efficacy or knowledge about putative active compounds or their mode of actions. *Resina Draconis* is a red resin from tree stem of *Dracaena cochinchinensis* (Lour.) S.C. Chen, growing in Yunnan and Guangxi provinces in China, belonging to theLiliaceae family*, genus Dracaena*. It was discovered by Cai and Xu [4] in 1979 that it could serve as a substitute for Sanguis Draconis, a precious crude medicine recorded in the official Chinese pharmacopoeia named as “dragon's blood” [5]. In Chinese medicine, *Resina Draconis* is a major component of the well-known hemostatic preparation “Yun Nan Bai Yao,” so, it is considered important for its potential application in Chinese medical practice. As a “panacea of blood activating” resin, RD has great medicinal value, and the main biological activity comes from phenolic compounds [6]. Pharmacological studies have showed that RD has positive effects on treatment of blood stasis syndrome, trauma, tumors, inflammation, gynecopathy, allergic dermatitis, and so on. It can promote blood circulation and serve as an antithrombotic, antioxidant, antiseptic, and anti-inflammation compound [7]. The ethanolic extracts of *Resina Draconis* possessed potential antithrombotic properties, affecting platelet aggregation and thus having anticoagulation activities [8]. All these studies indicate that *Resina Draconis* has enormous potential for further study.

It is well known that one kind of traditional Chinese medicine (TCM) usually contains a great number of components, which, all together, contribute to therapeutic effects. Despite that Chen et al. [9] have studied the wound-healing activity of dragon's blood (*Croton lechleri *sap), no scientific investigation is conducted on *Resina Draconis*'s wound-healing potential. Hence, in this study, we aimed to further investigate the wound-healing effects of RD using the excision and incision wound models, and to explore the possible mechanisms.

## 2. Materials and Methods

### 2.1. Preparation of Plant Extracts

Crude *Resina Draconis* was provided by the pharmacy department of No. 9 People's Hospital Affiliated to Shanghai Jiao Tong University School of Medicine. RD (30 g) was dissolved in absolute alcohol at room temperature for 48 h under shade and then was filtered. The solvent was concentrated under reduced pressure to ensure that no residual methanol was left behind furnishing a methanol extract. Normally, 4.57 g of dried powder can be obtained from 30 g of RD. The sample was stored at −80°C until used. The extract cream was formulated using ointment as the vehicle. The ointment consisted of propylene glycol : liquid paraffin (6 : 1) and applied topically onto the test animals. Extracts were prepared as 5% in the ointment.

### 2.2. Phytochemical Analysis

Preliminary phytochemical analysis was carried out using standard procedures to identify the constituents as described by Evans and Trease [10] and Harborne [11].

### 2.3. Experimental Animals

All study protocols were approved by the Shanghai Jiao Tong University Medical Center, Institutional Animal Care and Use Committee. Healthy Sprague-Dawley male rats weighing between 180 and 200 g were used. The rats were housed in polypropylene cage and maintained in standard laboratory conditions of temperature (22 ± 2°C) and light-dark cycle of 12 h : 12 h. They were maintained on standard pellet diet and provided with water *ad-libitum* throughout the experiment. At the end of the experiment the animals were sacrificed under anesthesia.

#### 2.3.1. Excision Wound Model

The anesthetized rats were inflicted with excision wounds as described by Morton and Malone [12]. The dorsal fur of the animals was shaved with an electric clipper, and the area of the wound to be created was outlined on the back of the animals with methylene blue using a circular stainless steel stencil. A full thickness of the excision wound of circular area of 254 mm^2^  and 2 mm depth was created along the markings with a surgical blade. The animals were randomly divided into 3 groups of 24 for each: Group I (control group) where animals were applied with simple ointment base [13]. Animals of Group II (standard group) were applied with a thin layer of moist exposed burn ointment (MEBO). Group III's (experimental group) animals were applied with a thin layer of the extract mixed with ointment. All vehicles were applied once daily, till the day of epithelization. The wound tissue was removed from control, MEBO, and RRDEE treated rats by sacrificing the animals on the 3rd, 7th, 11th, and 15th day after wound creation. Additionally, the rats of the three groups were maintained and treated as above for calculating of the rate of contraction and period of epithelialization.

#### 2.3.2. Incision Wound Model

The animals were randomly divided into three groups of six. Two 6 cm long paravertebral incisions were made using surgical blade (No. 15) through the entire thickness of skin at a distance of about 2 cm from the midline on each side of the depilated back of the rat. After the incision, surgical sutures were applied to the parted skin at intervals of one centimeter. The wounds were left undressed then ointment base, MEBO, and RDEE ointment were applied daily up to 10 days. When wounds were cured thoroughly, the sutures were removed on day 10 and the tensile strength of cured wound skin was measured.

### 2.4. Measurement of Wound Contraction

Wound margin was traced after wound creation by using transparent paper and the area was measured by graph paper. Wound contraction was measured every two days interval throughout the monitoring period

(1) Wound  contraction  (100%): =Initial  wound  size−Specific  day  wound  sizeInitial  wound  size×100%.

### 2.5. Epithelialization Period

The epithelialization time was measured from the initial day to the day when the scab fell off from the wound surface exclusive of leaving a raw wound behind [14].

### 2.6. Measurement of Tensile Strength

The force required to open the healing action is known as the tensile strength. It indicates how much the repaired tissue resists breaking under tension and may indicate in part the quality of the repaired tissue. The maximum load (tensile strength) tolerated by wounds was measured blindly on coded samples using a biomechanical analyzing instrument (Instron, Canton, MA, USA). Skin strips were stretched at a constant rate (1 mm/min) until disruption occurred. Wound breaking strength was expressed as the mean maximum level of tensile strength in newton (N) before separation of wounds.

### 2.7. Histopathological Studies

Granulation tissues from control and treatment groups were taken on the 3rd, 7th, 11th, and 15th day after wound creation. The specimens were formalin fixed and paraffin embedded according to the routine laboratorial techniques. Subsequently, serial 5 *μ*m thick sections was obtained and stained with Masson-trichrome (for detection of collagen fibers) and hematoxylin and eosin (H&E) (for general morphological observations). Slides were examined qualitatively under a light microscope, for collagen formation, fibroblast proliferation, angiogenesis, and granulation tissue formation [1, 15].

### 2.8. CD31 Immunohistochemistry Analysis and Microvessel Density (MVD)

Angiogenesis was assessed by CD31 (Epitomics, CA, USA) immunohistochemical in all the cases. Immunohistochemical staining was performed on paraffin sections using the SP method. After the sections were rinsed in distilled water, the endogenous peroxidase was inactivated with 3.0% hydrogen peroxide in distilled water for 10 minutes at room temperature. After rinsing the sections in phosphate-buffered saline (PBS, pH 7.4), the nonspecific binding site was blocked with 10% normal goat serum for 20 minutes at room temperature. The blocking serum was discarded, and then the primary antibodies were added directly. Rabbit anti-rat CD31 monoclonal antibody (Epitomics, CA, USA) was diluted to 1 : 500 in BSA. The sections were incubated with primary antibodies in a humid chamber at 4°C overnight. Sections were washed three times in phosphate buffer solution (PBS), and goat anti-rabbit polymer-peroxidase complex was added, and the sections were incubated for 30 minutes at room temperature. After rinsing with PBS, Streptavidin-horseradish peroxidase conjugate was added and the peroxidase activity was made visible with diaminobenzidine and counterstained with hematoxylin for 30 sec, then dehydrated, and mounted. Quantifications were performed by Image-Pro Plus 6.0 analysis system to calculate the integral optical density (IOD) of each field.

The microvessel density (MVD) was measured according to the method described by Zhen et al. [16]. Briefly, in areas of the most intense CD31 positive neovascularization, individual MVDs were made on a ×200 magnification field. Any endothelial cell or endothelial cell cluster was considered a single countable microvessel. MVD was expressed as the absolute number of microvessels per ×200 field for each case.

### 2.9. RNA Extraction and Quantitative Real-Time PCR

The expression patterns of TGF-**β**1 and VEGF of rat wound tissue on the 3rd, 7th, 11th, and 15th day after wound creation were analyzed by quantitative real-time PCR. Total RNA was extracted from the Granulation tissue sample with TRIzol (Invitrogen, Carlsbad, CA, USA) and then was further purified step using the RNeasy Mini Kit (Qiagen), in each case following the manufacturer's instructions. The concentration of the total RNA was detected. Total RNA (1 mg) in a 20 mL reaction volume was reverse transcribed into cDNA using the PrimeScript RT reagent kit (Takara, Dalian, China). Real-time PCR in 96-well optical plates was performed and analyzed with a Stratagene Mx3000P (Stratagene, La Jolla, CA, USA). The reactions were performed in a 20 mL volume using a SYBR Green reaction mix (Takara, Dalian, China) with 2 mL cDNA. The primers were: TGF-**β**1 forward: 5′-CGC AAC AAC GCA ATC TAT G-3′ and reverse: 5′-ACC AAG GTA ACG CCA GGA-3′; VEGF forward: 5′-TCA CCA AAG CCA GCA CAT AGG AGA-3′ and reverse: 5′-TTA CAC GTC TGC GGA TCT TGG ACA-3′; GAPDH forward: 5′-GAA CGG GAA GCT CAC TGG C-3′ and reverse: 5′-GCA TGT CAG ATC CAC AAC GG-3′. The thermal cycling consisted of denaturation for 30 sec at 95°C followed by 40 cycles of 30 sec at 95°C and 30 sec at 60°C. The threshold cycle (CT) values of target genes were normalized with GAPDH of the same sample and expressed as they were relative to controls.

### 2.10. Western Blot Analysis

Granulation tissues were homogenized in RIPA lysis buffer (150 mM NaCl, 1% Nonidet P-40, 0.1% SDS, 50 mM Tris-HCl pH 7.4, 1 mM EDTA, 1 mM PMSF, and 1 × Roche complete Mini Protease Inhibitor Cocktail). Protein concentration was determined using a BCA Protein Assay Kit. Equal amounts of protein were separated by 10% SDS gel electrophoresis (SDS-PAGE) under denaturing and nonreducing conditions and then transferred to a nitrocellulose membrane. The membrane was blocked with 5% nonfat milk in TBST at room temperature for 1 h and then incubated with anti-VEGF antibody (Abcam, UK, 1 : 200) at 4°C overnight. After washing in TBST, the blots were incubated with a horseradish-coupled secondary antibody. The signals were visualized using the enhancement system (ECL). GAPDH was used as the internal control and treated with the same protocol. The amount of proteins in gel slabs was quantified using a densitometer (Image Pro Plus 6.0 Media Cybernetics).

### 2.11. Statistical Analysis

The data were expressed as mean ± S.D. and performed using SPSS (Version19.0, Chicago, IL, USA). Significance was assessed by using the one-way ANOVA followed by *t*-test. Values were considered statistically significant when *P* value is less than 0.05.

## 3. Results

### 3.1. Phytochemical Analysis

The phytochemical analysis of the extract by qualitative method showed the presence of flavonoids, triterpenoids, steroids, cardiac glycosides, anthraquinones, carbohydrates, saponins, and phenols (Table 1).

### 3.2. Wound Contraction

**Figure 1:**
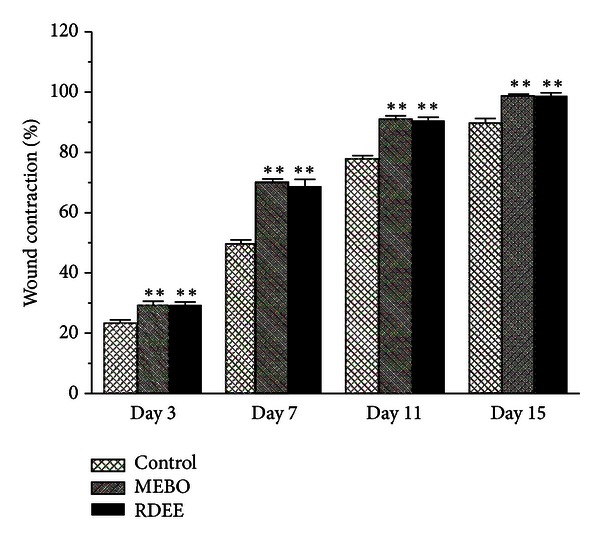
The rate of contraction in control, MEBO, and RDEE treated wound is shown here. Values are expressed as mean ± S.D. (*n* = 6 animals). **P < 0.01, versus control.

Wound contraction is an essential process in healing that leads to wound closure. The rate of contraction of the control group, MEBO group, and the RDEE treated group wounds is shown in (Figure 1). The results revealed that treatment with RDEE and MEBO resulted in much faster contraction of wound (*P* < 0.05).

### 3.3. Epithelialization Time

**Figure 2:**
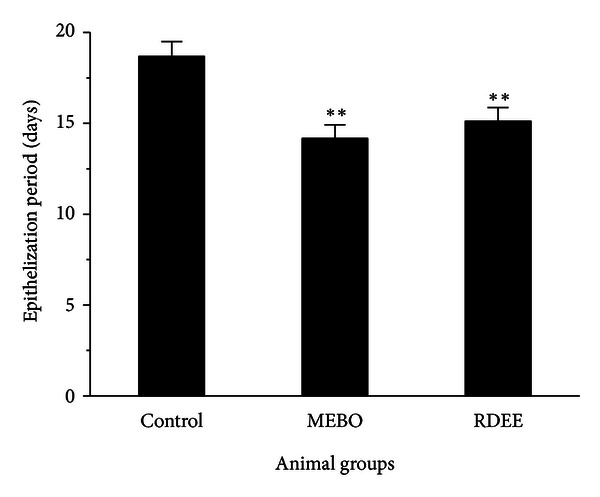
Period of epithelialization in control, MEBO, and RDEE treated wounds is shown. Values are expressed as mean ± S.D. (*n* = 6 animals). **P < 0.01, versus control.

The epithelialization time was measured from the first day. The epithelialization time was found to be significantly (*P* < 0.05) reduced in MEBO group and RDEE group as depicted in (Figure 2). Mean time to reepithelialization was 18.67 days (range, 18–20 days), control group; 14.16 days (range, 13–15 days), MEBO group; and 15.12 days (range, 14–16 days), RDEE group. There was no significant difference in the duration of wound healing between the groups treated with MEBO and RDEE; both healed by about 15 days. The control group, however, needed around 19 days to heal, about four days longer than the wound-healing time needed under MEBO and RDEE treatments.

### 3.4. Tensile Strength of Incision Wound Model

**Figure 3:**
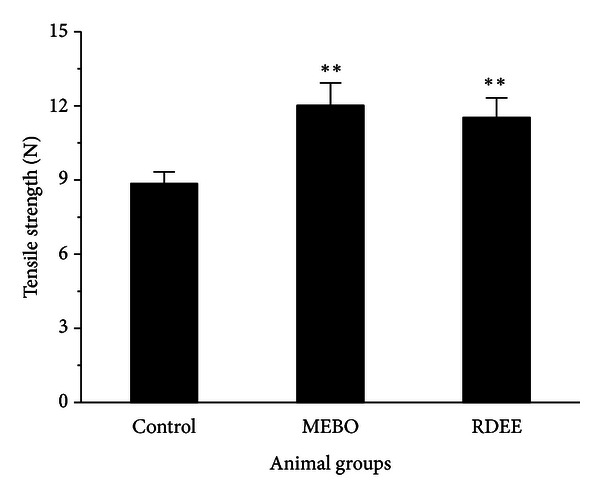
Tensile strength measurements of 10th day wound tissue of control, MEBO, and RDEE treated rats. Values are expressed as mean ± S.D. (*n* = 6 animals). **P < 0.01, versus control.

The results of the measurement of skin breaking strength on the 10th day after operation in incision wound-healing model were depicted in (Figure 3). A significant increase in the wound breaking strength (11.53 ± 0.79 N) was observed when compared with the controls (8.85 ± 0.48 N).

### 3.5. Histopathological Study

**Figure 4:**
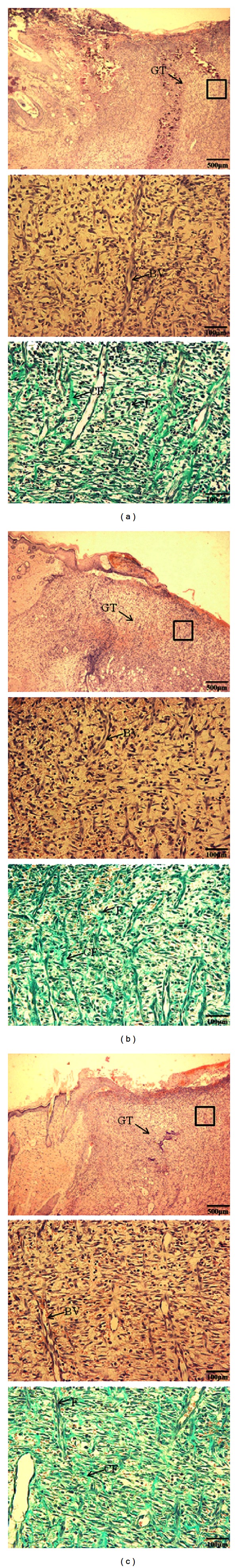
Photomicrograph of cutaneous wounds in rats at 7 days after wounding H&E stains. (a) Control group, (b) MEBO group, and (c) RDEE group. GT: granulation tissue; BV: blood vessel; F: fibroblasts cells; CF: collagen fibers.

**Figure 5:**
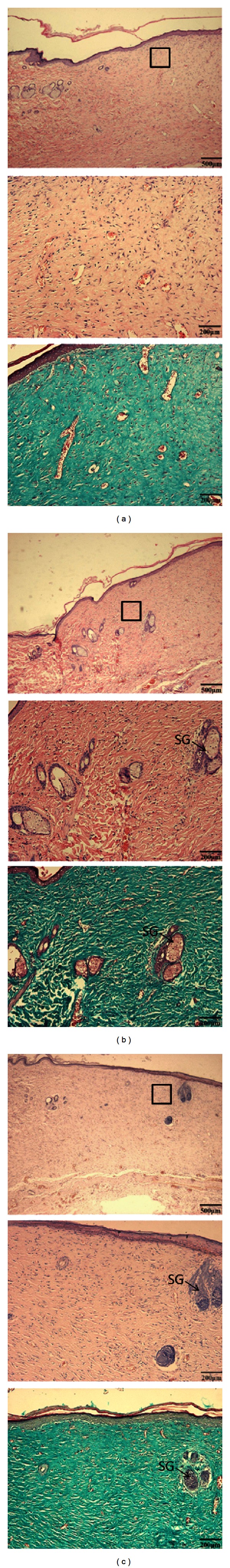
Photomicrograph showing histopathological changes of healed skin wounds on day 21 of postwounding (stained with hematoxylin-eosin and masson-trichrome). Collagen fibers were arranged more regularly and sparse than those of the scar tissue in the control group. (a) Control group, (b) MEBO group, and (c) RDEE group. SG: sebaceous gland.

Histopathological examinations of the healed wounds are shown in Figures 4 and 5. Two types of stains were used, Hematoxylin and Eosin (H&E) stains and Masson-Trichrome stains for general morphology. H&E stains collagen fibers pale pink, cytoplasm purple, nuclei blue, and red blood cells cherry red. Masson-Trichrome stains collagen blue, while cytoplasm, red blood cells, and muscle are stained red and is typically used to assess the advancement of collagen deposition during the formation of granulation tissue and matrix remodeling [17]. The blue colour staining intensity corresponds to the relative quantity of collagen fiber deposit, which reflects the process of synthesis and degradation and remodeling [18]. Histological sections of granulation tissue from RDEE treated rats showed more proliferating blood capillaries, collagen fibres, and fibroblasts cells (Figure 4), which was similar to the effect of MEBO treated group 7 days after wound creation, when compared with the control group. On the 21st day after wound creation, all groups of experimental rats showed complete epithelialization of the wound area. Masson staining revealed that the collagen bundles were thicker, denser disorganized, and more abundant in the control groups. By contrast, collage fibers were decreased and more regularly ranged in the groups treated with MEBO and RDEE. Otherwise, we observed sebaceous gland in the center (Figure 5).

### 3.6. Immunohistochemistry Analysis and MVD

**Figure 6:**
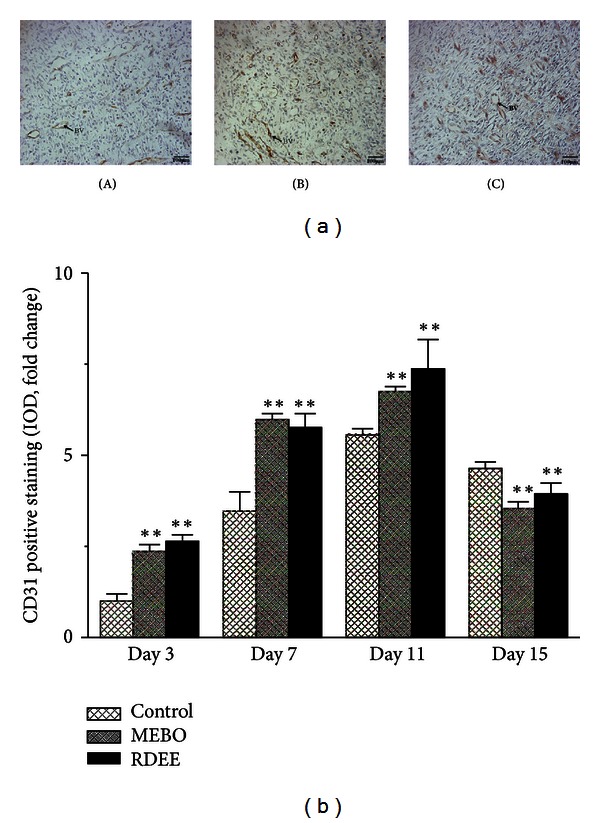
The histologic anti-rat CD31 staining of wound tissue samples. (a) Endothelial cells stained with the antibody were represented by brown colour. (A) Control group, (B) MEBO group, and (C) RDEE group. BV: blood vessel. (b) Quantitative analysis of the CD31 stain was calculated. All data were expressed as mean ± S.D. **P < 0.01, versus control.

Vascular endothelial cells were detected using a mouse anti-rat CD31 monoclonal antibody. CD31 expression was mainly present in the cytoplasm and membrane of endothelial cell or cluster. Being different from the control group, condensed, short, and twisted blood vessels were observed in the RDEE treated group (Figure 6(a)). In quantitative analysis, the intensity of CD31 was increased as compared with the control group on the 3rd, 7th, and 11th day (Figure 6(b)). The microvessel density (MVD) of the RDEE treated wounds was significantly higher than that of the control group (Table 2).

### 3.7. Gene Expression Analysis

**Figure 7:**
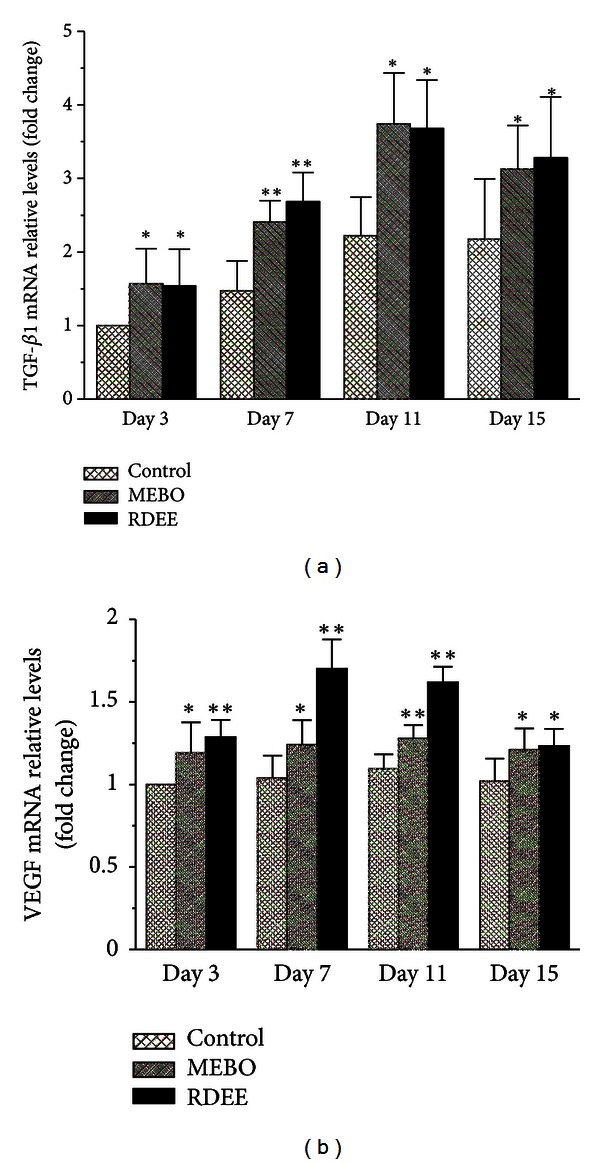
TGF-*β*1 and VEGF mRNA expression in cutaneous wounds. (a) TGF-*β*1; (b) VEGF. Normalization relative to GAPDH was performed. Results presented in bar graph are the mean ± S.D. *P < 0.05, **P < 0.01, versus control.

To investigate the molecular mechanism of RDEE-induced wound-healing activity, the expression levels of related genes were examined. TGF-*β*1 and VEGF are the major genes that are generally involved in wound healing. Following several days of treatment, a significant increase in expression of TGF-*β*1 and VEGF in the wound tissue of extract treated rats was noted, as compared with the control animals (*P* < 0.05). The results showed a day-dependent effect on TGF-*β*1 and VEGF mRNA expression. The mRNA expression levels of TGF-*β*1 of the three groups increased from day 3 after injury reaching pick levels at day 11 and then it declined constantly; the mRNA expression of VEGF was also increased from day 3 and correspondingly peaked at day 7 (Figures 7(a) and 7(b)). TGF-*β*1 and VEGF mRNA expression significantly correlated with the wound contraction.

### 3.8. Protein Levels by Western Blotting

**Figure 8:**
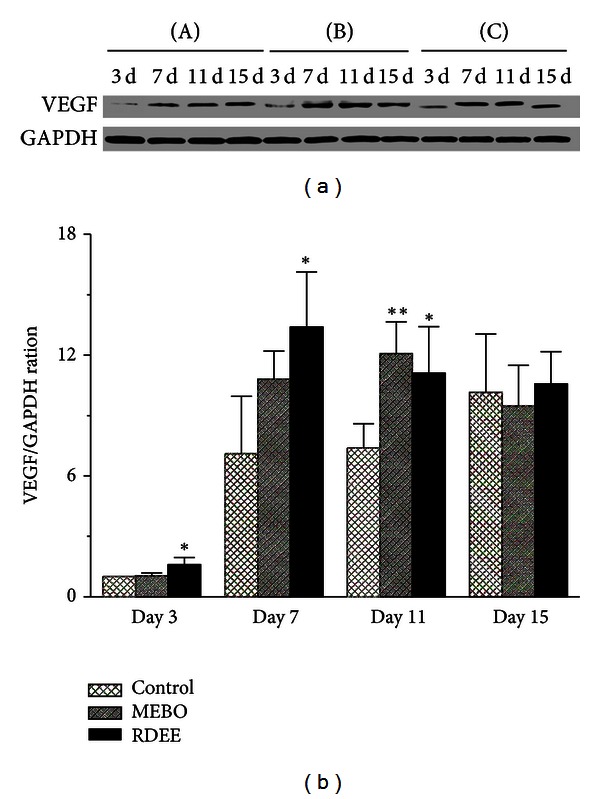
(a) Western blot results for vascular endothelial growth factor (VEGF) protein in the three groups. (b) Quantification of densitometry analysis of protein levels. All data were expressed as mean ± S.D. *P < 0.05, **P < 0.01, versus control.

We further examined the protein levels of VEGF in the granulation tissues of rats from the three groups by Western blotting. Western blot analysis showed an upregulated expression of VEGF in RDEE treated group as compared with the control group. On the 3rd, 7th, and 11th day after wound creation, the protein levels of VEGF were significantly increased in RDEE treated group (*P* < 0.05) (Figure 8(a)). Quantified protein level and the fold changes were shown in Figure 8(b).

## 4. Discussion

Wound healing is a complicated process. The aim of wound healing is to promote rapid wound closure and recover functional properties. Hence in this study, excision and incision wound models were used to evaluate the effects of RDEE on wound healing. The significant reduction in wound size and mean epithelization time as well as the higher expression of growth factors in the RDEE treated group as compared with those from vehicle group corroborate with the histopathological findings of increased epithelization activity, angiogenesis, and higher collagen fibers formation. These findings imply that RDEE promoted wound-healing activity via angiogenesis, collagen deposition, epithelization, and wound contraction.

Excisional and incisional wounds are the two main wound models in wound research which allowed the determination of the wound-healing phases. The excisional wound is found to be more suitable for histological evaluation due to the broader morphological changes occurring during the process of wound healing. Wound contraction is an essential process in healing which leads to wound closure. Thus, visible appearances and measurements of wound contraction become reliable parameters in macroscopic evaluation for wound healing [19]. This study showed that RDEE significantly stimulated the contraction of wounds as seen from the percentage of wound contraction (Figure 1). Reepithelialization is important, as it restores the integrity of the skin, making it less vulnerable to infection. The extract-treated animals showed a decreased time to epithelialization (Figure 2) compared with the control group. In incision wound, the increase in tensile strength of RDEE treated wounds may be due to the increase in collagen concentration and stabilization of the fibres [20]. Since incision wound treated with RDEE showed greater tensile strength, it might be speculated that it not only increased collagen synthesis per cell, but also aided in cross-linking of the protein. Histological analysis further revealed that topical application of RDEE significantly increased the fibroblast growth, collagen synthesis, and the healing process.

Histological evaluation showed that healing process of the wounded tissue in RDEE treated group was comparably close to the reference MEBO treated group, whereas significant difference was observed in negative control group. Granulation tissue primarily contains fibroblasts, collagen fibres, very less edema, and newly generated blood vessels, which were observed in RDEE treated group of animals (Figure 4). This histopathological observation provided additional evidence for the experimental wound-healing studies based on the contraction value of wound areas and the measurement of tensile strength. Enhanced healing activity has been attributed to increased collagen deposition and angiogenesis [21]. Collagen plays a central role in the healing of wounds, and it is a principal component of connective tissue and provides a structural framework for the regenerating tissue. Histopathological study showed better proliferation of collagen fibers in the RDEE group compared with the control group (Figure 4). In addition, the slim, defined, and well-organized collagen fibers in the RDEE treated group reinforce the improved quality of the final remodeling of the wound (Figure 5). Angiogenesis during wound repair serves the dual function of providing the nutrients required by supplying essential nutrients and oxygen to the wound site and promoting granulation tissue formation [22]. Histological evaluation showed an increase in the number of blood vessels in the granulation tissue of the rats treated with RDEE. Enhanced expression of CD31 as revealed through immunohistochemistry in RDEE treated group might be responsible for this activity. In the present study, the treatment group was found to increase angiogenesis as evidenced by MVD (Table 2).

Many types of cytokines and growth factors are responsible for inflammation, reepithelialization, the formation of granulation tissue, and neovascularization during the wound healing process [23]. Transforming growth factor beta is an important growth factor that regulates different cellular functions in all phases of wound healing. TGF-*β*1 produced by fibroblasts as a multifunctional cytokine acts on these cells [24] and enhances granulation tissue formation and collagen formation in wound-healing process [25]. TGF-*β*1 has also been reported to encourage wound contraction through its direct induction of alpha smooth muscle actin expression in fibroblasts [26]. Otherwise, VEGF appears to be a key factor in pathological situations such as tissue repair, which involves neovascularization and increased vascular permeability. VEGF improves angiogenesis during the process of wound healing by stimulating the migration of endothelial cells through the extracellular matrix [27]. VEGF also has been demonstrated to mediate angiogenic activity during the proliferative phase of wound healing [28]. In addition, VEGF mediates vascular hyperpermeability and promotes the secretion of active growth factors and cytokines necessary for wound repair [29]. The animals treated with RDEE significantly enhanced the expression of VEGF, which is the most potent angiogenic factor during wound healing, thereby, stimulating the formation of new blood vessels [30]. In the present study, our results showed that the expression of VEGF was significantly higher in the RDEE treated group and peaked on the 7th day (Figures 7(b) and 8).

The wound-healing property of RD may be attributed to the phytoconstituents present in the resina, and the quicker process of wound healing could be a function of either the individual or the additive effects of the phytoconstituents. In ethnopharmacological studies, the effects of dragon's blood on various biological activities, such as attenuate visceral nociception, antiviral, antibacterial, and antifungal have been reported [31, 32]. Our preliminary phytochemical screening of ethanolic extract of RD showed the presence of flavonoids, triterpenoids, steroids, cardiac glycosides, anthraquinones, carbohydrates, saponins, and saponins (Table 1). Recent studies with other plant extracts have shown that phytochemical constituents such as flavonoids [33] and triterpenoids [34] are known to promote the wound-healing process mainly due to their astringent and antimicrobial properties, which appear to be responsible for wound contraction and increased rate of epithelization. Previous studies have revealed that the resin is rich in flavonoids, sterols, and terpenoids [35, 36]. Possibly, the wound-healing action of RD may probably be due to the presence of phytoconstituents in the plant or could be a function of either the individual or the additive effects of the phytoconstituents; however, further phytochemical studies are needed to isolate the active compound(s) responsible for these pharmacological activities.

## 5. Conclusion

We have shown that the RDEE facilitates wound healing in the experimental animal model. There is a need for further studies in order to isolate the active ingredients in the plant that are responsible for its biological activities and to elucidate the mechanisms of actions of these active ingredients.

## Figures and Tables

**Table 1:** Results of the phytochemical analysis of RD extract.

Constituents	Result
Cardiac glycosides	+
Flavonoids	+
Catechin tannins	−
Triterpenes	+
Carotenoids	−
Anthraquinones	+
Carbohydrates	+
Saponins	+
Phenols	+
Steroids	+

+: presence; −: absence.

**Table 2:** The comparison of MVD (mean ± S.D.) between each group.

Group	MVD (mean ± S.D.)			
	3 d	7 d	11 d	15 d
Control	20.00 ± 2.28	24.67 ± 4.50	46.00 ± 5.87	55.00 ± 4.05
MEBO	32.00 ± 3.35**	60.83 ± 3.54**	69.00 ± 3.58**	44.33 ± 4.13**
RDEE	35.33 ± 3.88**	62.50 ± 3.56**	72.33 ± 4.32**	51.50 ± 8.36

All values are expressed as the mean ± S.D. Means labeled with superscripts were significantly different. **P < 0.01, versus control.
